# Increase in mucormycosis hospitalizations in southeastern Brazil during the COVID-19 pandemic: a 2010-2021 time series

**DOI:** 10.1590/0037-8682-0333-2022

**Published:** 2023-02-20

**Authors:** Ivan Lira dos Santos, Carolina Specian Sartori, André Giglio Bueno, Elisa Teixeira Mendes

**Affiliations:** 1 Pontifícia Universidade Católica de Campinas, Faculdade de Medicina, Campinas, SP, Brasil.; 2 Pontifícia Universidade Católica de Campinas, Hospital da PUC-Campinas, Departamento de Infectologia, Campinas, SP, Brasil.; 3 Pontifícia Universidade Católica de Campinas, Programa de pós-graduação em Ciências da Saúde, Centro de Ciências da Vida, Campinas, SP, Brasil.

**Keywords:** Hospitalization trend, Mucormycosis, COVID-19

## Abstract

**Background:**

Mucormycosis is a severe invasive fungal disease. During the coronavirus disease 2019 (COVID-19) pandemic, outbreaks have been reported worldwide, but epidemiological studies are still scarce in Brazil.

**Methods:**

We conducted a time-series cohort hospitalization study (2010-2021) in southeastern Brazil.

**Results:**

There were 311 cases (85 during the pandemic), with significant (*P* < 0.05) involvement of patients older than 40 years (84%), white patients (78%), rhinocerebral site (63%), and São Paulo State residents (84%).

**Conclusions:**

Mucormycosis hospitalizations were highly prevalent. Further studies are needed to assess the burden of COVID-19 on mucormycosis in Brazil.

Mucormycosis is a severe and lethal fungal disease. Urgent surgical intervention and antifungal therapy are lifesaving^1^. The causal pathogens are fungi from the mucorales order with the main reported genera *Rhizopus* spp, *Mucor* spp, and *Rhizomucor* spp^1^. Clinically, infection is classified based on anatomic localization, such as cutaneous, disseminated, gastrointestinal, pulmonary, and rhinocerebral^2^. Classical risk factors associated with mucormycosis include uncontrolled diabetes mellitus, direct inoculation, corticotherapy, immunosuppression, solid organ transplantation, onco-hematological disease, and immunotherapy (especially with tocilizumab) used in the management of severe coronavirus disease 2019 (COVID-19)^1^ ^,^ ^3^. In the last 2 years, there has been a notable incidence of COVID-19-associated mucormycosis (CAM). Prolonged hospitalization and high-dose corticosteroid use in patients with severe COVID-19 are the main factors for this increase^4^ ^-^ ^7^. In Brazil, epidemiological studies are still scarce, with only five case reports and technical notes^8^ ^-^ ^13^. 

This was a retrospective time-series study that evaluated the trend of mucormycosis hospitalization rates in southeastern Brazil from 2010 to 2021. This period was separated into pre-and-pandemic onset, 2010-2019 and 2020-2021, respectively. We exported secondary data from the *Sistema de Informações Hospitalares* (SIH) [Brazilian Hospital Information System] and selected patients with the 10th International Classification of Diseases (ICD): B46.0-B46.9 in at least one of the diagnosis fields provided through the hospital admission authorization (AIH) from 2010 to 2021. To compare the two periods, 2010-2019 and 2020-2021, we created a time-series graph (Figure 1) and used the *chi-square* test for categorical variables and the *Kruskal*-*Wallis* test for continuous variables, with *P* ≤ 0.05. The variables evaluated were sex (male, female), age group (< 40 years and > 40 years), skin color (white, brown, black, indigenous, yellow, and unanswered), state of residence (SP, MG, ES, and RJ), anatomical site (cutaneous, gastrointestinal, pulmonary, and rhinocerebral), bed occupation (surgical, clinical, and other), days of hospitalization, intensive care unit (ICU) admission, emergency hospitalization, onco-hematological and transplant disease, and discharge or death outcome. For geographical visualization (Figure 2), we built maps of hospitalization cases according to the patient’s municipality of residence. Analyses were carried out using R-Studio (https://posit.co/products/open-source/rstudio/) software with the aid of the following packages: *microdatasus* for data collection, tidyverse and dplyr for data processing, tableone for frequency tables, geobr for maps, and ggplot2 for graphics**.**

**FIGURE 1:**
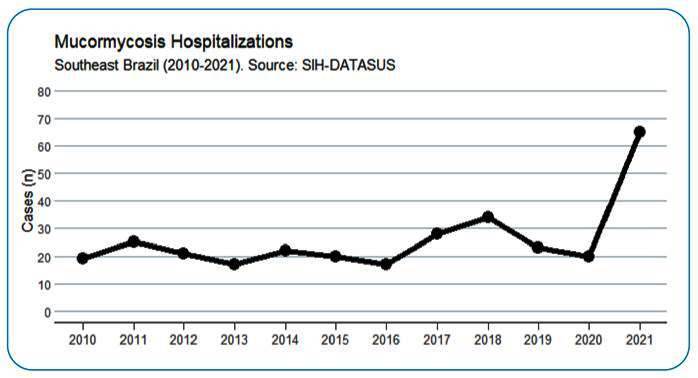
Time series of mucormycosis hospitalizations in southeastern Brazil (2010-2021). Source: Sistema de Informações Hospitalares (SIH) [Brazilian Hospital Information System].

**FIGURE 2:**
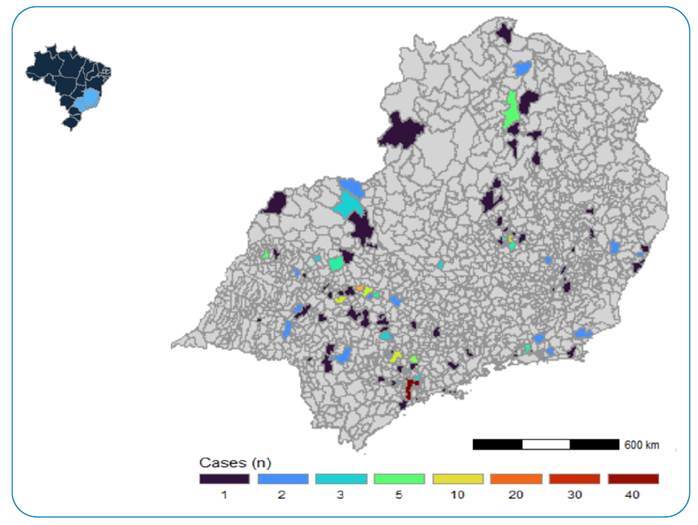
Map of mucormycosis hospitalizations according to the municipality of residence in southeastern Brazil (2010-2021). **Source:** Sistema de Informações Hospitalares (SIH) [Brazilian Hospital Information System].

**Ethical approval:** Studies using an anonymous secondary public database did not require approval from the Brazilian National Commission for Ethics in Research.

Hospitalization rates were higher during the pandemic period, especially in 2021. There were 311 patients within the 11 years and 85 cases during the pandemic period (Figure 1). Most patients were men (62%), with white skin (67%), with a median age of 54 years and an interquartile range (IQR) of 39-66 years. The in-hospital lethality was 11.9%. We observed a high frequency of cases in the state (213) and city of São Paulo (46) (Figure 2). Belo Horizonte presented the highest frequency in the state of Minas Gerais, with nine cases; Rio de Janeiro and Espírito Santo’s cases were not restricted to their capitals (Figure 2). 

Table 1 describes the main clinical and epidemiological characteristics of the mucormycosis cases during the two periods. The most common anatomical site was the rhinocerebral region (42%). Thirteen percent of the hospitalizations required ICU, the median hospital stay was 9 days (IQR: 4-20), and 10% of the patients had onco-hematologic or transplant diseases.

**TABLE 1:** Time series of mucormycosis in southeastern Brazil, cohort 2020-2021 vs. cohort 2010-2019.

	Pre-pandemic (2010-2019)	Pandemic (2020-2021)	Total	*P*-value*
**n**	226	85	311	
**Sex**				0.646
Female	88 (38.9)	30 (35.3)	118 (37.9)	
Male	138 (61.1)	55 (64.7)	193 (62.1)	
**Median Age [IQR]**	54 [36-64]	54 [44-69]	54 [39-66]	0.307
**Age Group**				**0.038**
> 40	161 (71.2)	71 (83.5)	232 (74.6)	
< 40	65 (28.8)	14 (16.5)	79 (25.4)	
**Skin Color** ^†^				**0.020**
White	119 (62.0)	62 (77.5)	181 (66.5)	
Black and Brown	73 (38.0)	18 (22.5)	91 (33.5)	
**State of Residence**				**0.005**
São Paulo	142 (62.8)	71 (83.5)	213 (68.5)	
Minas Gerais	57 (25.2)	10 (11.8)	67 (21.5)	
Rio de Janeiro	20 (8.8)	4 (4.7)	24 (7.7)	
Espírito Santo	7 (3.1)	0 (0.0)	7 (2.3)	
**Anatomic Site**				**0.026**
Rhinocerebral	49 (36.8)	22 (62.9)	71 (42.3)	
Pulmonary	54 (40.6)	11 (31.4)	65 (38.7)	
Cutaneous	26 (19.5)	2 (5.7)	28 (16.7)	
Gastrointestinal	4 (3.0)	0 (0.0)	4 (2.4)	
**Specialty**				0.590
Surgical	57 (25.2)	24 (28.2)	81 (26.0)	
Clinical	151 (66.8)	56 (65.9)	207 (66.6)	
Others	18 (8.0)	5 (5.9)	23 (7.4)	
**Median hospital stays [IQR]**	9 [4-20]	9 [3-20]	9 [4-20]	0.446
**Intensive Care Unit**	31 (13.7)	9 (10.6)	40 (12.9)	0.586
**Urgency**	160 (70.8)	65 (76.5)	225 (72.3)	0.393
**Onco/Hematological/Transplant**	25 (11.1)	6 (7.1)	31 (10.0)	0.402
**Death**	29 (12.8)	8 (9.4)	37 (11.9)	0.526

* For categorical variables, the chi-square test is used, and the Kruskal-Wallis test is used for continuous variables. IQR stands for interquartile range. † yellow n = 1, excluded from the Table.

There was a statistically significant difference in the proportional distribution of cases between the different types of anatomical sites considered (*P* = 0.026). Rhinocerebral mucormycosis was more frequent in the second period, and the pulmonary clinical form was less frequent. During the pandemic period, patients over 40 years of age (*P* = 0.038) and whites (*P* = 0.020) were significantly more prevalent. Lethality, need for ICU admission, and days of hospital stay were similar between the two periods.

We found 85 mucormycosis hospitalizations during the pandemic period, a significantly higher rate than that in the pre-pandemic period. This finding was similar in other regions of the world, especially in India^5^ ^,^ ^6^, as well as in the United States, Iran, France, and Mexico^4^.

To date, five cases of CAM have been reported in Brazil, one in Manaus^10^, and four in São Paulo^9^ ^,^ ^11^ ^,^ ^12^. All patients had rhinocerebral infection, four had a previous diagnosis of diabetes, and three had severe COVID-19 requiring mechanical ventilation, with one death. Recently, the Brazilian Health Surveillance Agency (ANVISA) published a report warning of an increase in cases of mucormycosis in the pandemic context^13^. Geographically, in our study, cases were concentrated in large centers, such as São Paulo statewide, São Paulo city, and the capital of the state of Minas Gerais (Belo Horizonte). Large referral centers drain more critical cases and have a higher diagnostic capacity. In addition, the concentration of mucormycosis cases in these centers is associated with a higher number of patients with COVID-19.

A published review of 80 CAM reports and case series also described rhinocerebral infections as the most common site of infection^4^. The authors highlighted that the COVID-19 pandemic poses a substantial risk for invasive fungal infections, especially because of the increased number of intensive care patients, long hospitalizations, antibiotic use, and systemic corticosteroids^8^ ^,^ ^11^ ^,^ ^12^ ^,^ ^14^. Additionally, corticosteroids were the only therapy with an impact on mortality in the first wave, and their prolonged use significantly altered glycemia in patients with diabetes.

We assessed low lethality in our data (11.6%) compared with the literature (39.2-45.7%)^6^ ^,^ ^15^, which can be attributed to the SIH system structure. SIH is the official Brazilian hospitalization database with in-hospital services for reviewing and validating electronic medical records. A system that counts hospitalizations rather than patients per unit potentially masks the high mortality reported in the literature. As for limitations, we used a secondary data source containing only public SUS hospitalizations without direct microbiological information, even though mucormycosis diagnosis can only be performed using microbiological or anatomopathological analysis. 

Mucormycosis is an emerging invasive fungal infection in the COVID-19 era. Our study has identified a recent increase in the occurrence of mucormycosis in southeastern Brazil. The alert from ANVISA may have played a role in improving diagnosis. Further studies are needed to assess the impact of COVID-19 on mucormycosis occurrence in Brazil.
